# Prevalence and characteristics of smoking cigarette and narghile among Syrian refugee parents in Ontario, Canada

**DOI:** 10.1371/journal.pgph.0003176

**Published:** 2024-05-03

**Authors:** Hala Tamim, Aliya Beyhum, Aseel Alzaghoul, Durmalouk Kesibi, Baraa Alghalyini, Khalid Yunis

**Affiliations:** 1 Department of Kinesiology and Health Science, York University, Toronto, Ontario, Canada; 2 Department of Archeology, University of Cambridge, Cambridge, United Kingdom; 3 Division of Social and Transcultural Psychiatry, McGill University, Montreal, Quebec, Canada; 4 Family Medicine, Alfaisal University, Riyadh, Saudi Arabia; 5 Department of Pediatrics and Adolescent Medicine, American University of Beirut Medical Center, Beirut, Lebanon; Pai Chai University, KOREA, REPUBLIC OF

## Abstract

This study aimed to explore the prevalence and associated sociodemographic, migration, and health-related factors of smoking cigarettes and narghile among Syrian refugee parents resettled in Ontario, Canada since 2015. A total of 540 Syrian refugee parents, with at least one child less than 18 years of age, were interviewed. Multivariate logistic regression analysis was performed to assess the independent relationship between each of the associated sociodemographic, migration, and health-related factors and smoking cigarettes and narghile. The overall prevalence of smoking was 43% (cigarette = 22%, narghile = 25.6%). The average number of cigarettes smoked per day was 15.4 (SD = 10.1). With regard to smoking narghile, 18.2% of the sample smoked daily, while 35.5% and 52.9% smoked at least once weekly and occasionally, respectively. Results from multivariate logistic regression analysis showed that more fathers were at increased odds of smoking cigarettes than mothers OR (95% CI) = 6.6 (3.7–11.9), while no such difference was found for narghile smokers OR (95% CI) = 1.5 (0.9–2.6). Results showed that smoking cigarettes was associated with lower education levels and being Muslim, whereas smoking narghile was associated with younger age and alcohol use. Poor perceived mental health was significant/approached significance for both types of smoking. Greater understanding of smoking patterns of resettled refugees is needed to direct healthcare providers to offer targeted interventions for those who are most at risk.

## Introduction

Culture plays an important role in smoking patterns and prevalence, which can vary widely between different countries and cultural norms. In Syria, like the majority of Arab and Middle Eastern countries, the waterpipe or the *narghile*, is a common social pastime. Narghiles may vary in shape and size, but their mechanics are similar. The process involves burning charcoal to heat a flavored tobacco mixture that is placed on an aluminum foil on top of the smoking instrument. The smoke then moves down and bubbles through the water before traveling through an attached smoking pipe to be inhaled by the smoker [1]. Healthwise, frequently smoking narghile causes similar long-term health effects as smoking cigarettes. Smoking narghile for an hour involves approximately 200 inhalations, while smoking one cigarette involves 20 inhalations [2]. Like smoking cigarettes, narghile has been linked to increased incidence of cancer, especially lung and esophageal cancers [3]. Despite evidence that the health effects of narghile are as harmful as smoking cigarettes, it appears that countries have less policies, educational awareness campaigns, and laws regulating its use. Moreover, some narghile venues and products in many developed countries are exempt from tobacco control policies [1].

A study conducted in 2006 in Aleppo, Syria’s largest city prior to the Civil War, revealed that overall, there was a higher prevalence of daily cigarette smoking (29.0%) than daily narghile smoking (1.0%) [4]. However, a recent study conducted in 2019 in Syria during the Civil War, revealed a lower overall rate of daily cigarette smoking (6.4%) but a higher rate of daily narghile smoking (11.8%), with significantly higher rates for cigarette smoking and narghile smoking in males compared to females [5]. While both types of smoking have harmful effects on one’s health, smoking narghile is culturally acceptable in Syria because it is perceived as a social leisure activity rather than a personal addiction to manage stress. A study by Hammal et al., (2008), showed that the key difference between the two types of smoking habits is the attitudes towards them. *Narghile* smokers described the waterpipe as a social activity and part of Syrian cultural heritage. They believed that the habit was harmless and thought that it fostered “togetherness” [6]. On the other hand, cigarette smokers used cigarettes as a way to manage stress and described it as mundane and a personal addiction. They also acknowledged health concerns that came with the habit [6].

Indeed, the prevalence and method of smoking, such as cigarettes, narghile, or vaping, vastly differ depending on customs, tradition and region from one country to another [7].

As of 2019, approximately 63,938 Syrian refugees have been granted legal residence in Canada as part of the *Syrian Refugee Resettlement Initiative* [8], that prioritizes the resettlement of vulnerable refugees, especially families and women with children [9]. According to a report by the Pan-Canadian Health Inequalities Reporting Initiative, individuals who either belong to the lowest income group, are unskilled workers, or did not complete high school are more likely to have higher rates of smoking than their compatriots, revealing the double jeopardy of the "smoking inequality"–this is when disadvantaged and vulnerable groups have a higher risk of smoking and are exposed to subsequent detrimental effects [10]. This was demonstrated in a recent study published in 2021 that examines the trends of cigarette smoking amongst Syrian refugees during their first two years in Canada. The study reported a 27% prevalence of the sample smoking cigarettes on a regular basis [11], a rate higher than the reported Canadian average of 15.6% [12]. Unfortunately, little is known about narghile smoking amongst the Syrian refugee population in Canada. Given the growing number of Syrian refugees and the external factors that may lead to increased smoking, this study focuses on resettled Syrian parents in Ontario. Moreover, varying degrees of societal acceptance or rejection concerning smoking behaviours among refugees highlight the importance of understanding and addressing such intricacies for enhanced integration of individuals in new host countries. Thus, studying the patterns of smoking cigarettes and narghile can be useful for refugee resettlement agencies and national policies. The objective of the study was to assess the prevalence of smoking cigarettes and narghile among Syrian refugee parents in Ontario and identify its associated factors.

## Methods

The study design was cross-sectional in nature. A total of 540 Syrian refugee parents were recruited and interviewed between March 3, 2021, and March 10, 2022. The inclusion criteria required the participants to be a Syrian refugee parent residing in Ontario, Canada, with at least one child under the age of 18 at the time of interview and resettled in Canada after 2015. Participants were recruited and interviewed through telephone interviews in order to comply with social distancing guidelines due to the COVID-19 pandemic. The participants were recruited through convenience sampling with the help of organizations including Access Alliance Multicultural Health and Community Services and the Arab Community Centre of Toronto. Research assistants who could read, write, and speak in Arabic, specifically in the Syrian dialect, administered the survey. Each participant received a $20 honorarium for their participation in the study.

The status and frequency of cigarette and narghile smoking were collected from all participants, with questions specifically designed to capture their current habits. Socio-demographic, migration, and health-related factors were also collected from all participants. Socio-demographic characteristics considered for the study included gender, age (≤ 35, 36–45, >45), number of children (≤2, 3–4, >4), highest level of education (≤ elementary, secondary-high school/diploma, university), religion (Muslim, non-Muslim), employment status, and self-perceived socioeconomic status (SES) measured by the question, “In your current condition, here in Canada, would you say most people would categorize a household like yours as” (lower income, lower middle income / middle income, upper middle income / upper income). Migration-related factors included country of residency before Canada (Jordan, Lebanon, Turkey, other) having resided in a refugee camp, year of arrival to Canada (during or before 2016, after 2016), and perceived level of Canadian languages- English or French (excellent / good, fair / poor, very poor / not at all). Health-related factors addressed alcohol consumption and self-rated mental-health (excellent, very good / good, fair / poor).

Descriptive statistics was performed to assess the level of cigarettes and/or narghile smoking and describe characteristics of the study participants. Logistic regression models were conducted to assess the association between each of the socio-demographic, migration, and health-related factors, and smoking cigarettes and/or narghile. Two multivariable logistic regression models were performed; one with smoking cigarettes as the outcome variable and the other with smoking narghile as the outcome variable. Each of the models included all of the socio-demographic, migration, and health-related factors. The model for cigarette smoking adjusted for smoking narghile, whereas the model for smoking narghile adjusted for smoking cigarettes. Odds Ratios (ORs) and 95% Confidence Intervals (95% CIs) were reported. All regression models were adjusted for the clustering effect of belonging to the same family. All analyses were conducted using the Statistical Package for the Social Science (SPSS, version 26.0). The project was approved by the Research Ethics Board at York University (Certificate # e2019-128) and was conducted in accordance with the ethical standards of the Helsinki Declaration. Informed consent was obtained from all participants. To comply with social distancing recommendations due to the COVID-19 pandemic, questionnaire surveys were conducted remotely over the phone. Participants were informed about the objectives of the study and the voluntary nature of their participation, with the option to opt-out at any point without any repercussions. Individuals who decided not to participate, were treated with the same respect as those who participated, thus ensuring ethical standards and fairness. Before the administration of the questionnaire, the research assistant sent electronically to participants a soft copy of the consent form. The research assistant then went over the consent form and answered any questions the participants had. The research assistant then recorded the audio of the participants’ oral consent.

## Results

Table 1 shows the descriptive statistics of the study sample and the bivariate association of smoking cigarette and/or narghile with the different socio-demographic, migration-related, and health-related factors among study participants. The average age of the 540 Syrian refugee parents recruited for the study was 39.7 years (SD = 7.3) with 60.9% of participants being mothers, 65.6% being unemployed and 48.9% having had 2–3 children. More than half of participants arrived in Canada during or before 2016 (57.8%) and 55% ranked their socioeconomic status in Canada as lower income (36.9%). The overall prevalence of smoking cigarettes or narghile was 43.9%; 22.0% smoked only cigarettes, 25.6% smoked only narghile, and 3.7% smoked both cigarettes and narghile. The average number of cigarettes smoked per day was 15.4 (SD = 10.1) with 43.7% of smokers being light smokers (smoked ≤14 cigarettes per day) and 52.9% heavy smokers (smoked ≥15 cigarettes pers day). Narghile was smoked daily, weekly (at least once), and occasionally by 18.1%, 35.5%, and 46.4% of the study participants, respectively.

**Table 1:** Relationship between smoking and socio-demographic, migration-related, and health-related factors among study participants.

	Total	Cigarettes Smokers			Narghile Smokers		
	n (%)	%	OR (95% CI)	P value	%	OR (95% CI)	P value
**Socio-demographic characteristics**							
**Gender**							
Mothers	329 (60.9)	10.0	1		26.1	1	
Fathers	211 (39.1)	40.8	6.2 (3.9–9.8)	**<0.001**	24.6	0.9 (0.6–1.3)	0.716
**Age**							
≤ 35	153 (28.3)	12.4	0.3 (0.1–0.7)	0.002	32.7	3.9 (1.9–7.7)	**<0.001**
36–45	275 (50.9)	25.1	0.8 (0.5–1.4)	0.569	27.3	2.8 (1.5–5.4)	**0.001**
> 45	11 (20.6)	27.9	1		11.7	1	
**Number of children**							
≤2	170 (31.5)	18.8	1		35.9	1	
3–4	264 (48.9)	19.7	1.1 (0.6–1.8)	0.670	23.5	0.5 (0.3–0.8)	0.005
>4	106 (19.6)	33.0	2.2 (1.2–4.0)	0.004	14.2	0.2 (0.1–0.5)	**<0.001**
**Education**							
≤ Elementary	101 (18.7)	43.6	4.9 (2.6–8.9)	**<0.001**	16.8	0.5 (0.2–1.0)	0.066
Secondary-high school/ Diploma	278 (51.5)	18.0	1.2 (0.7–2.2)	0.339	27.7	1.0 (0.6–1.6)	0.892
University	161 (29.8)	15.5	1		27.3	1	
**Religion**							
Muslim	340 (63.0)	27.1	2.7 (1.7–4.5)	**<0.001**	20	0.4 (0.3–0.6)	**<0.001**
Non-Muslim	192 (35.6)	13.5	1		35.9	1	
**Employment**							
Yes	186 (34.4)	27.4	1.5 (1.0–2.3)	0.038	31.2	1.5 (1.0–2.3)	0.037
No	354 (65.6)	19.2	1		22.6	1	
**Self-perceived SES**							
Lower income	199 (36.9)	27.1	2.0 (0.8–5.0)	0.137	18.1	0.4 (0.2–0.9)	0.029
Lower middle / middle income	297 (55)	19.5	1.3 (0.5–3.3)	0.542	30.0	0.8 (0.4–1.7)	0.654
Upper middle / upper income	39 (7.2)	15.4	1		33.3	1	
**Migration-related factors**							
**Country of residence (before Canada)**							
Jordan	106 (19.6)	29.2	1		17.9	1	
Lebanon	221 (40.9)	19.5	0.5 (0.3–0.9)	0.021	28.5	1.8 (1.0–3.3)	0.038
Turkey	97 (18.0)	27.8	0.9 (0.4–1.6)	0.785	18.6	1.0 (0.5–2.1)	0.902
Others	116 (21.5)	15.5	0.3 (0.1–0.7)	0.005	32.8	2.2 (1.1–4.4)	0.012
**Lived in a refugee camp**							
Yes	64 (11.9)	29.7	1.6 (0.9–2.9)	0.092	17.2	0.5 (0.2–1.1)	0.119
No	476 (88.1)	21.0	1		26.7	1	
**Year of Arrival to Canada**							
During or before 2016	312 (57.8)	21.5	1		23.7	1	
After 2016	222 (41.1)	22.5	1.0 (0.6–1.5)	0.968	27.9	1.2 (0.6–1.8)	0.368
**Canadian language**							
Excellent / good	195 (36.1)	15.9	1		29.2	1	
Fair / poor	270 (50.0)	20.7	1.3 (0.8–2.2)	0.175	25.2	0.8 (0.5–1.2)	0.354
Very poor / not at all	75 (13.9)	42.7	3.9 (2.1–7.1)	**<0.001**	17.3	0.5 (0.2–0.9)	0.047
**Health-related factors**							
**Alcohol drinking**							
Yes	78 (14.4)	23.1	1.0 (0.5–1.8)	0.915	47.4	3.2 (1.9–5.4)	**<0.001**
No	462 (85.6)	21.9	1		21.9	1	
**Self-rated Mental health**							
Excellent	64 (11.9)	25	0.3 (0.3–1.5)	0.571	21.9	0.7 (0.3–1.5)	0.460
Very good/ good	279 (51.7)	16.8	0.5 (0.3–0.8)	**0.002**	25.8	1.0 (0.6–1.4)	0.827
Fair/ poor	197 (36.5)	28.4	1		26.4	1	
**Smoking Cigarettes**							
Yes	119 (22.0)				16.8	0.5 (0.3–0.8)	0.014
No	421 (78.0)				28.0	1	
**Smoking narghile**							
Yes	138 (25.6)	14.5	0.5 (0.3–0.8)	0.014			
No	402 (74.4)	24.6	1				

Table 2 shows the results of the multivariable logistic regression models. Cigarette smoking was significantly higher among fathers compared to mothers OR (95% CI) = 6.6 (3.7–11.9) and among participants whose highest level of education was elementary, compared to those with a university degree OR (95% CI) = 4.9 (2.6–8.9). Contrastingly, smoking narghile was significantly associated with age. The results showed that when compared to participants > 45 years of age, those who were ≤ 35 and participants between 35–45 years of age were at increased odds of smoking narghile with OR (95% CI) being 6.3 (2.8–14.4) and 4.2 (2.0–8.7) respectively. Additionally, religion was significantly associated with cigarette smoking among Muslims than non-Muslims OR (95% CI) = 3.3 (1.5–7.5) but not associated with narghile smoking. With regards to health-related factors, alcohol consumption was significantly associated with increased narghile use OR (95% CI) = 3.2 (1.7–6.1) but was not associated with smoking cigarettes OR (95% CI) = 1.5 (0.7–3.5). Meanwhile, those with a mental health rating of very good to good showed significantly decreased odds for smoking cigarettes OR (95% CI) = 0.5 (0.3–0.9), compared to participants who rated their mental health between fair to poor. The relationship between self-rated mental health and smoking narghile approached significance when participants who rated their mental-health as good to very good and excellent were at decreased odds for smoking narghile compared to participants with a fair to poor mental-health rating (ORs (95% CI) being 0.7 (0.4–1.1) and 0.5 (0.2–1.1) respectively). Finally, results of the multivariate logistic regression model showed that participants who smoked narghile were less likely to smoke cigarettes OR (95% CI) = 0.5 (0.3–1.0) and participants who smoked cigarettes were less likely to smoke narghile OR (95% CI) = 0.5 (0.3–1.0).

**Table 2:** Results of multivariable logistic regression for the association between smoking and socio-demographic, migration-related, and health-related factors among study participants.

	Cigarettes		Narghile	
	Adjusted OR (95%CI)	p-value	Adjusted OR (95%CI)	p-value
**Socio-demographic characteristics**				
**Parents**				
Mothers	1		1	
Fathers	6.6 (3.7–11.9)	**<0.001**	1.5 (0.9–2.6)	0.161
**Age**				
≤ 35	0.9 (0.4–2.2)	0.876	6.3 (2.8–14.4)	**<0.001**
36–45	1.4 (0.7–2.7)	0.287	4.2 (2.0–8.7)	**<0.001**
> 45	1		1	
**Number of children**				
≤2	1		1	
3–4	0.6 (0.3–1.2)	0.142	0.8 (0.5–1.3)	0.350
>4	0.5 (0.2–1.3)	0.160	0.6 (0.3–1.6)	0.335
**Education**				
≤ Elementary	4.9 (1.9–12.8)	**0.001**	1.0 (0.4–2.5)	0.992
Secondary-high school/ Diploma	1.5 (0.7–3.0)	0.284	1.4 (0.8–2.4)	0.255
University	1		1	
**Religion**				
Muslim	3.3 (1.5–7.5)	**0.004**	0.8 (0.4–1.5)	0.412
Non-Muslim	1		1	
**Employment**				
Yes	1.6 (0.9–3.0)	0.132	1.2 (0.7–2.0)	0.563
No	1		1	
**Self-perceived SES**				
Lower income	1.1 (0.4–3.3)	0.9	0.6 (0.2–1.5)	0.267
Lower middle/ middle income	1.1 (0.4–3.3)	0.8	1.0 (0.4–2.3)	0.995
Upper middle–upper income	1		1	
**Migration-related factors**				
**Country of residence (before Canada)**				
Jordan	1		1	
Lebanon	0.9 (0.4–1.9)	0.765	1.3 (0.6–2.7)	0.473
Turkey	0.8 (0.3–1.6)	0.474	1.3 (0.6–2.9)	0.508
Others	0.6 (0.3–1.6)	0.343	1.7 (0.8–3.8)	0.199
**Lived in a refugee camp**				
Yes	0.8 (0.4–1.7)	0.563	0.8 (0.3–1.8)	0.574
No	1		1	
**Year of Arrival to Canada**				
During or before 2016	1		1	
After 2016	1.1 (0.6–1.9)	0.818	1.0 (0.6–1.7)	0.892
**Canadian language**				
Excellent / good	1		1	
Fair / poor	1.1 (0.6–2.1)	0.832	1.2 (0.7–1.9)	0.529
Very poor / not at all	1.5 (0.6–4.0)	0.366	1.4 (0.6–3.6)	0.458
**Health-related factors**				
**Alcohol drinking**				
Yes	1.5 (0.7–3.5)	0.310	3.2 (1.7–6.1)	**<0.001**
No	1		1	
**Self-rated Mental-health**				
Excellent	0.6 (0.2–1.3)	0.195	0.5 (0.2–1.1)	0.086
Very good/ good	0.5 (0.3–0.9)	**0.030**	0.7 (0.4–1.1)	0.124
Fair/ poor	1		1	
**Smoking Cigarettes**				
Yes			0.5 (0.3–1.0)	0.053
No			1	
**Smoking narghile**				
Yes	0.5 (0.3–1.0)	0.037		
No	1			

## Discussion

The present study examined the prevalence of cigarette and narghile smoking and its’ associated socio-demographic, migration, and health-related factors among Syrian refugee parents resettled in Ontario, Canada since 2015. A high prevalence and different predictors for smoking cigarettes and narghile were observed in the sample. Being a male and having lower education were factors that were significantly associated with smoking cigarette smoking, while younger age and alcohol use were significantly associated with smoking narghile. While none of the migration-related factors were found to be significant predictors of either type of smoking, poor perceived mental-health was significant/approached significance for both types of smoking. Knowledge of refugee smoking prevalence and risk predictors can better help foresee which groups are more at risk and in need for intervention programs, especially considering the large number of refugees Canada welcomes every year.

There have been several efforts and awareness campaigns in Canada to continue reducing smoking rates in the country [12]. On average, a pack containing 20 cigarettes could cost $12.49 [13] in Ontario, where the hourly minimum wage is $15.50 [14]. While a session of narghile in a café in Canada costs between $12-$15 [15]; in Lebanon, it can cost up to $7.92 [16], which is expensive given their minimum wage being $800 per month [17]. However, the price of narghile smoking is much cheaper if prepared at one’s home [18]. Overall prevalence of smoking reported in the present study was 43%, with 22% smoking cigarettes and 25.6% smoking narghile. These results are significantly higher than rates reported in Canada– 19.1% of Ontarians over the age of 12 report smoking in the past 30 days, of whom 15.6% smoke cigarettes and 0.8% smoke narghile [12]. Further, results of the present study are consistent with findings from the Syrian population in 2019, that reported overall smoking rates of 38% (cigarette = 16.4%, narghile = 29.35%) [5], and a recent study conducted by Oda (2021) et al., where smoking cigarettes was reported among 27% of Syrian refugees in their first two years of resettlement in Canada [11].

Indeed, the high prevalence of smoking among Syrian refugees can be attributed to multiple factors such as exposure to war-induced stressors, traumatic events, substandard quality of life, and limited opportunities [19]. Results of the present study indicated that Syrian refugee fathers were 7 times at increased odds of smoking cigarettes compared to mothers OR (95% CI) = 6.6 (3.7–11.9), while no significant difference in narghile use was found between father and mother participants OR (95% CI) = 1.5 (0.9–2.6). These results are consistent with a recent study that was conducted in Syria that found males to be at increased odds of smoking cigarette than females OR (95% CI) = 4.8 (3.3–6.7), whereas the magnitude of the association for narghile smoking among men compared to women was significantly lower OR (95% CI) = 1.4 (1.0–1.8) [5]. Similarly, a study of government-assisted Syrian refugees in Ottawa reported that the prevalence of tobacco use was 60.3% among males and significantly higher than females [20]. Consistent results for cigarette smoking were found in a study of several Middle Eastern countries including Lebanon, Jordan, and Palestine, with the latter showing very high odds of smoking associated with males OR (95% CI) = 66.9 (29.3–153.1). However, results varied for narghile, showing lower odds of narghile use amongst men in Lebanon OR (95% CI) = 0.49 (0.37–0.64), but higher in Jordan and Palestine (OR (95% CI) = 1.92 (1.40–2.621); OR (95% CI) = 2.69 (1.85–3.92) respectively) [21]. The results of these studies may be partly explained by the fact that cigarette smoking is not very socially acceptable for females in Middle Eastern countries, while narghile smoking is considered acceptable in social culture [7] and is usually practiced with family and friends [22]. Typically, it is the duty of females in the household to prepare the narghile, and in its preparation they must inhale it to make sure it is well prepared [23]. In two different studies, females have reported that the exposure of having to prepare the narghile led them to acquiring the habit of smoking narghile [24, 25]. This could partly explain the social acceptance of narghile for females as compared to cigarette smoking.

The current study showed that participants in the youngest age group (≤35 years of age) were significantly at increased odds for smoking narghile compared to older participants. This finding is consistent with previous literature from the Middle East [26, 27] that also showed higher rates of narghile use among younger age groups. These finding could be explained by the fact that smoking narghile seems to be perceived as less harmful [22, 28–30] and is associated with less stigmas than smoking cigarettes among young individuals [28]. Narghile is also more affordable compared to cigarettes, which may appeal to the younger population [31]. In addition, increased odds of smoking cigarette were observed in lower education categories, a finding that is in agreement with other literature [27, 32]. Individuals with higher education are more aware of the harmful effects of smoking [33] and may have more access to health services [32], which discourages them against smoking or encourages them to quit smoking. Moreover, the results revealed that Muslims were at higher odds of smoking cigarettes compared to non-Muslims. Similarly, a study of Muslims in the United States reported elevated rates of tobacco use among Muslims than the general population. A plausible explanation could be attributed to the cultural acceptance of tobacco consumption in certain Muslim-majority countries [34]. Another reason could be that smoking is considered an acceptable practice for Muslims. Unlike alcohol consumption, there is no explicit prohibition of tobacco use in the Islamic law sources, resulting in a limited influence of religious beliefs on efforts to quit smoking [35].

The results of this study showed a significant association between alcohol drinking and narghile smoking. These results are consistent with several studies on youth in the US which found narghile users to have a significantly higher association [28, 31, 36, 37] and increased severity and frequency with alcohol consumption [28] compared to non-users. Both narghile and alcohol are associated with social contexts [31], and previous research has showed that substance use tends to co-occur [28]. Additionally, a study conducted among US students found that 70% of the sample reported drinking alcohol while smoking narghile with the justification that alcohol intensifies the relaxing and pleasurable effects of narghile [37].

Results from the current study showed that parents with poor mental health smoked more cigarettes and narghile than those with good mental health. Consistently, several studies on youth and adults have found significant associations between poor mental-health and increased use of narghile [38–42] and cigarette smoking [43, 44] compared to non-users of each method. The association between mental-health and smoking seems to be bidirectional. It is postulated that individuals use smoking as a method to alleviate their symptoms of stress, depression, and anxiety [45]. However, nicotine, the main addictive ingredient in cigarettes and narghile, increases physiological arousal and is thought to stimulate anxiety [45]. Furthermore, nicotine withdrawal in periods between smoking sessions may lead to symptoms of depression [46].

Although this is the first study, to our knowledge, that assesses the prevalence and characteristics of smoking narghile among Syrian refugee parents in Canada, several limitations must be noted. First the cross-sectional design of the study may involve a reverse causality between odds of smoking cigarettes and narghile, and the socio-demographic, migration, and health-related variables. Second, there is the possibility of selection bias as a result of the voluntary participation in the study. Third, information bias may be present as all responses were collected by self-report. Fourth, the results may also be subject to confounding biases such as participants’ smoking habits in Syria and before migrating to Canada, which was not collected for the present study. Additionally, information on participants’ living areas, such as community type, urban or rural setting was not gathered as part of this study.

This study provides an understanding of the prevalence and risk factors associated with resettled refugees that make them at an increased risk group for smoking cigarettes or narghile. The high rates of smoking among this population calls for targeted interventions towards refugees, specifically refugees from the Middle East. Different predictors were found for cigarette and narghile smoking; healthcare provides should focus their interventions on individuals with these predictors, so as to achieve better outcomes. Efforts should be made to target mental health and reduce migration stress for refugees as a first step in the intervention process. Also, special attention should be given to narghile use amongst younger age groups, given the higher odds of its use within this age group. Lastly, future qualitative studies are essential to explore the sociocultural aspects that impact refugees’ integration experiences to offer a more holistic understanding of their challenges and adaptations.
